# S138. AN INVESTIGATION INTO THE ASSOCIATION BETWEEN EXPOSURE TO PRENATAL STRESS AND RISK OF PSYCHOSIS IN OFFSPRING

**DOI:** 10.1093/schbul/sby018.925

**Published:** 2018-04-01

**Authors:** Ross Brannigan, Antti Tanskanen, Matti O Hunnunen, Mary Cannon, Finbarr Leacy, Mary Clarke

**Affiliations:** 1 Royal College of Surgeons in Ireland; 2 Karolinska Institutet; 3 National Institute for Health and Welfare

## Abstract

**Background:**

Existing literature suggests that prenatal stress may be a risk factor for offspring psychiatric disorders. For example, large ecological studies have found that those exposed to stressors during gestation, such as war and famine, have a twofold increase risk of schizophrenia, as well as an increased risk for other affective disorders. Similarly, it was found that exposure to stressful events during pregnancy, such as the death of a relative during first trimester, increases the odds of the offspring developing schizophrenia in adulthood. In this study, our aim was to assess in a birth cohort, whether those who were exposed to prenatal stress were at higher odds for developing psychosis and other psychiatric disorders.

**Methods:**

Using the Helsinki temperament cohort, a yearlong birth cohort with data collected from pregnancy onwards, logistic regressions were run examining perceived prenatal stress as a risk factor for psychosis and other psychiatric disorders. The exposure (prenatal stress) was measured using prenatal questionnaires which were given to pregnant women at antenatal clinic visits if birth was expected between 1st July 1975 and 30th June 1976. Psychiatric outcomes were assessed using linkage between the Finnish population register and the Finnish hospital discharge register in 2005.

**Results:**

In total, 3660 pregnant women submitted at least one prenatal questionnaire with the mean number of prenatal questionnaires submitted per woman being 6. At the point of register access, 226 individuals had either an ICD 8, 9 or 10 diagnoses, 72 diagnosed with a psychosis disorder. It was found that those exposed to prenatal stress were at a greater risk of developing psychosis (OR = 1.54, 95% CI = 0.78 – 3.05).

**Discussion:**

Our findings are in line with the current literature indicating a higher risk of psychosis among those exposed to perceived prenatal stress.

